# Alcohol-Exposed Pregnancy Risk, Mental Health, Self-Understanding, and Relational Connections Among Urban Native American Young Women During the COVID-19 Pandemic

**DOI:** 10.3390/ijerph22030358

**Published:** 2025-02-28

**Authors:** Sara M. London, Caitlin T. Howley, Michelle Sarche, Carol E. Kaufman

**Affiliations:** 1 College of Education, University of Washington, Seattle, 1410 NE Campus Parkway, Seattle, WA 98195, USA; 2 James Bell Associates, Inc., 2000 15th Street North, Suite 100, Arlington, VA 22201, USA; howley@jbassoc.com; 3 Buffett Early Childhood Institute, University of Nebraska, 2111 S. 67th Street, Omaha, NE 68106, USA; michellesarche@nebraska.edu; 4 Centers for American Indian and Alaska Native Health, Colorado School of Public Health, University of Colorado Anschutz Medical Campus, 13055 E. 17th Avenue, Aurora, CO 80045, USA; carol.kaufman@cuanschutz.edu

**Keywords:** native WYSE CHOICES, alcohol-exposed pregnancy, COVID-19, mental health, identity, contraceptive use, sexual choices

## Abstract

The COVID-19 pandemic had a disproportionate impact on American Indian and Alaska Native (“Native”) communities, including factors impacting alcohol-exposed pregnancy (AEP) risk. This is especially true for young Native women in urban settings, where over 70% of the population resides, yet their experiences are rarely accounted for in research. We conducted remote in-depth interviews from March to May 2022, roughly concurrent with the Omicron surge and relaxed lockdown measures, with a subsample of 15 urban Native young women ages 16–20 who were participating in a national randomized controlled trial of an AEP preventive intervention. Participants were asked how the pandemic affected their use of alcohol, sexual health, mental health, and relationships. A qualitative analysis revealed diverse experiences during the pandemic. While some participants experienced greater risks for AEP due to increased alcohol use and reduced access to birth control, other participants drank less alcohol and had greater access to birth control. Additionally, while some participants faced mental health challenges due to isolation and relational strains that emerged during the pandemic, others found the pandemic to be a time that afforded self-reflection, self-development, and a deepening of relationships.

## 1. Introduction

American Indian and Alaska Native (hereafter referred to as “Native”) communities were disproportionately impacted by the COVID-19 pandemic [1,2,3]. Historical trauma and long-standing inequities in Native communities, including weak public funding and infrastructure and limited access to health care, housing, and potable water contributed to higher risk for COVID-19 morbidity and mortality [4,5,6]. Research regarding the impact of the pandemic has largely focused on reservation and other tribal land-based communities. However, more than 72% of Native people reside in urban settings [7]. While urban-residing Native people make up the majority of the Native population, they make up a small fraction of most urban communities where they reside. Many urban Native people are tightly connected to tribal communities and cultural practices [8], commonly traveling between urban areas and tribal reservation communities to participate in family events or important cultural celebrations and ceremonies. However, urban Native people are likely to experience different cultural, social, and economic impacts of the pandemic compared to those living in tribal reservation settings [9,10], and those differences may be critical with respect to alcohol-exposed pregnancy (AEP) risk.

According to the Centers for Disease Control, any sexually active person who is able to become pregnant, drinks alcohol, and does not use effective contraception is at risk for an AEP [11]. For Native communities, the prevention of AEP risk among youth has long been a priority given the opportunity to leverage culturally protective strengths at a time when data show that risky behaviors begin to emerge [12,13]. Protective factors are traditional beliefs about respecting one’s body and holding it sacred that are passed on to Native youth by families, elders, and coming-of-age ceremonies. Placing Native youth at risk, however, are patterns of drinking and sexual health that, combined, create vulnerability to AEP. Past research has shown that Native youth are more likely to have consumed alcohol in their lifetime (72.1%) compared to White youth (48.9%) and to have engaged in binge drinking (15% vs. 7%) [14,15,16]. Research has also shown that the rate of lifetime alcohol use among Native girls (ages 12–18), in particular, is significantly higher (71%) than rates among White girls (56%) or Native boys (58.4%) [16]. These data must be understood within the context of the intergenerational trauma and structural racism that Native people face. In a systematic review of trauma in relation to substance use among Native people, findings showed that substance use disorders (SUDs) were linked to lifetime trauma and post-traumatic stress disorder [17]. Another study found trauma exposure rates were higher among women (74%) versus men (25.9%) [18]. Among families with intergenerational trauma, such as the legacy of boarding schools, there was a correlation with SUDs in their communities [19]. Additionally, research has found that Native women residing on rural reservations identified their exposure to violence as a cycle passed down from past generations and from themselves to their children [20]. Structural racism also influences the health and wellbeing of Native people with research showing that SUDs are linked to the effects of historical and current racial discrimination [21].

Additional studies have shown that alcohol consumption is associated with risky sexual activity [22,23]. Pregnancy rates for Native youth are disproportionately higher than any other ethnic group, and unplanned pregnancy among Native youth is two to three times greater than among non-Hispanic White teenagers [24]. Little research exists on patterns of AEP among urban Native young women in particular. What data do exist indicate that they may be at the same or greater risk for alcohol use and sexual risk compared to their non-Native counterparts [25]. One study found alcohol/drug use and sexual risk behavior rates to be twofold higher than among their reservation-based peers [26].

With the abrupt onset of the pandemic in 2020, many factors were likely to have influenced patterns of alcohol use and sexual risk. Data from the early stages of the pandemic in the general youth population indicated an increased frequency of alcohol use, particularly among females whose use of alcohol was higher than males’ use [27], and among Native youth, higher levels of current use and binge drinking when compared to all other race and ethnic groups except White and multiracial non-Hispanic youth [28]. Coupled with declines in access to effective contraception among women [29], it is possible, but not known, that AEP risk, and among urban Native young women—often with limited culturally appropriate local resources—any increased risk may have been especially elevated. Moreover, the research base on risk-taking or coping and its association with conditions arising from the pandemic remains thin. Isolation disrupted education and access to reproductive health care, and an unstable economy contributed substantially to pandemic stress [30,31,32,33,34], even while research with reservation-based Native youth points to varied profiles of risk and resilience during the pandemic [35]. Still, we know little about how these contextual changes in urban Native young women’s lives influenced their AEP-related risk-taking. Similarly, we know little about other relevant impacts on health and wellbeing or how they found resilience and strength in their families and communities to overcome pandemic hardships. This paper endeavors to fill this gap for urban Native young women’s experiences during the pandemic.

## 2. Materials and Methods

Native WYSE (Women, Young, Strong, and Empowered) CHOICES was a randomized controlled trial (RCT) designed to evaluate the effectiveness of a culturally tailored AEP prevention program translated for mobile app delivery among urban Native young women ages 16-20 nationally [36]. Supplemental funding awarded during the COVID-19 pandemic permitted the collection of additional quantitative and qualitative data to assess study participants’ COVID-19-related experiences, including COVID-19-related shifts in behavior, attitudes, and perceptions affecting AEP risk. This paper reports on the additional qualitative data that were gathered. The Native WYSE CHOICES RCT and supplemental work benefitted from the guidance of an Urban Community Advisory Board comprising urban Native health leaders, researchers with experience in AEP prevention and Native health research, and Native youth [36,37]. The principal investigators’ university institutional review board reviewed and approved this study.

*Design*: To assess participants’ pandemic-related experiences, including those affecting AEP risk, qualitative in-depth interviews (IDIs) were conducted by phone in 3 intervals between March 2022 and November 2023. A sample size of 16 was planned for each interval to capture breadth and depth of the pandemic experience with sufficient saturation. The current paper is an analysis of IDIs conducted during the first interval, which occurred between March and May 2022. This set of interviews is uniquely positioned to capture participants’ experiences during the period of the pandemic when the Omicron variant continued to surge, even as lockdown requirements were easing.

*Recruitment*: IDI participants were recruited from existing enrollees in the Native WYSE CHOICES RCT by email, text messaging, and phone calls. Recruitment into the RCT occurred remotely through social media, email blasts, organizational newsletters, and referrals by other participants, friends, and family. RCT participants were eligible if they were ages 16–20, assigned female at birth, lived in an area with a population of 50,000 or more outside of reservation or other tribal lands in the lower 48 United States, were not pregnant or breastfeeding, and had a smartphone. After completing an eligibility survey via Qualtrics, prospective RCT participants were contacted by study staff to confirm urban residence and rule out fraudulent responders by verifying prospective participants’ identity through confirmation of date of birth and other personal information that would be difficult for a fraudulent responder to know. Within the informed consent obtained from all RCT study participants, participants could indicate their interest in participating in an IDI after completing all RCT activities and follow-up surveys. To achieve a set of IDI participants with diverse perspectives and experiences, prospective IDI participants were identified through a purposive sampling of the RCT participants who indicated an interest in an IDI. Such purposive sampling is widely used in qualitative research to identify and select participants who can provide rich data [38]. Purposive sampling is also a strategy for effectively gathering critical information from hard-to-reach or uniquely positioned populations [38]. Our purposive sampling strategy was designed to include individuals with a range of both AEP-related experiences and COVID-19-related experiences based on their responses to RCT baseline surveys. This strategy yielded a sample that included individuals who were and were not at risk for an AEP as well as individuals with diverse pandemic-related experiences, including those with and without friends/family who experienced COVID-19 infections and those who had and had not been vaccinated. This recruitment strategy yielded 15 participants at interval 1, the majority of whom (n = 13) were not at risk for an AEP based on their responses to the RCT baseline in which they indicated no risky drinking in the last 30 days (i.e., no report of heavy (8+ drinks in a week) or binge (4+ drinks on a single occasion) drinking) and no possibility of becoming pregnant in the last 30 days due to abstinence or use of effective birth control if not abstinent.

*Interviews*: Although each IDI participant had already consented to an IDI as part of their initial consent into the RCT, the consent form was reviewed before each IDI began. Each IDI lasted approximately 1 h. All were conducted by telephone and recorded to minimize technology or bandwidth challenges; all recordings were professionally transcribed. Each participant received an electronic gift card valued at USD 40 for completing an IDI.

*Measures*: The IDI interview guide covered the following topics: perceived changes in participants’ personal choices or changes in alcohol use, sexual activity, and contraceptive use during the pandemic; how the pandemic shaped participants’ employment and educational opportunities or aspirations; and resources, including those that were culturally based, that were instrumental in supporting participants through the pandemic.

*Data Analysis*: Two coders analyzed transcript data using the mixed methods software program Dedoose [39]. A combination of inductive and deductive coding [40,41] approaches was employed. The primary coder began data analysis by carefully analyzing the transcripts through inductive and deductive coding. Then, the codes were categorized and reorganized into thematic representations. A secondary coder coded the transcripts using the same process. To solidify the reliability of this study’s findings, both coders discussed the emergent themes they discovered, and each coded a subset of the transcripts and reached an interrater reliability of >95% (Kappa) [42].

## 3. Results

In the following, we describe key themes that emerged from our interviews, summarized in Table 1. While the focus of the interviews was on AEP-related experiences, including quotes demonstrating each point, we also include sections foregrounding mental health, identity, and relationships that this sample of Native young women described in the interviews.

### 3.1. Patterns of Alcohol Use

When participants reflected on their choices about alcohol use, some shared that the pandemic was *not* the most important factor affecting their decisions to drink; instead, growing older and going to college were the events affecting their choices to drink. For others, the stressors of the pandemic played a central role in either increasing or decreasing alcohol use. Isolation, poor mental health, and boredom were commonly mentioned as additional key factors. Participants also shared that alcohol was easier to obtain during the pandemic due to friends who were of legal drinking age being bored during the pandemic:

*[It has been easier to get alcohol during the pandemic …] Because you have other friends who are actually 21 who are bored, too, and can’t really do anything, so then they want something to do, so they’ll easily just get it for you.*

A common reflection among participants was how the alcohol abuse they witnessed in their families and communities impacted the choices they made about their drinking, including during the pandemic. One participant, for example, shared that the pandemic was a time of actively healing the stressors in their life, including those stemming from exposure to alcohol and drugs earlier in life, and that as part of their healing, they decided to drink significantly less.

*I think that I was exposed at a young age to alcohol and drugs, and I feel like I wasn’t raised to kind of learn what the after-effects were of these things. And it was to the point where I felt like I had to drink or to smoke or whatever because it was the cool thing to do. And it wasn’t even that much. It was never like I did it every day, or I felt like I had to always be doing it or be high or whatever. I think it was more because I didn’t know what else to kind of help with my anxiety with people. And then, now, having the help that I need with my anxiety and my depression, I’ve never really had any want…anymore to do drugs or to be drinking … And I also think because I had such a massive influence with my dad drinking and smoking, I never knew that it was the wrong thing. I just knew that that was normal, [inaudible] that did that. And I never saw that as something that was even a thing that-I knew that a lot of people in my community, in the Native American community that I was from, smoked and drank alcohol, but we weren’t really raised with the mindset of this isn’t what you should be doing. We were raised with this is what’s going to kind of happen to you. Just expect this … It’s like there’s no other way to be kind of. This is what is the norm, and yeah. And then it’s realizing, “Actually, I don’t have to live my life like this.”*

All participants were asked to reflect on the influences that family, friends, and community had on their choices about drinking. Some participants discussed the positive influences family had, while others shared how being exposed to the effects of alcohol on their families motivated them not to drink or to drink less.

*But my parents are both completely straight edge. My dad, this year, I think, started to actually drink and smoke weed and smoke cigars, which he, my whole life, was avidly against because both of my parents’ parents, my grandparents, were alcoholics and had a lot of substance abuse issues and struggles with that, so I was raised by my parents to be like, “You never drink. You never smoke. Never do anything. If you try it once, you’ll be addicted. It’s in your genes. Don’t do it.” So, there was a lot of shame around especially weed, which we might talk about after, I don’t know, but that was something that I’ve struggled with, just a lot of shame around it. But because I think my parents were very athletic and very physically active and never put any of that in their bodies, I kind of grew up with this association of alcohol is poison, and I don’t want to do that to my body even if it sometimes feels okay.*

Some participants shared that their friends significantly affected their drinking habits, stating that they surrounded themselves with like-minded friends.

*Well, my best friend, actually, she doesn’t drink very much either, so it kind of works out. And so having her kind of always I don’t know- So we will go out, and we’ll hang out with our friends and stuff, and she always manages to have a good time without drinking. And so having that kind of influence in my life is also really good because it’s like, you know what I’m saying, you could always have you can have fun without drinking, and you can have fun.*

In contrast, several participants shared that their friends did *not* affect their drinking habits; they had their personal preferences, and they were not swayed by their friends’ decisions about using alcohol.

*I mean, I don’t know. I feel like, for me, it’s always a very personal decision. I never feel pressured by my friends to drink, and I mean that. I mean, I’m sure if I’m with a lot of people, I’ll probably drink, but if I don’t want to, I won’t.*

Many participants also noted the role of their Native community’s traditional value to stay clean and sober. Others shared the deep effects of historical trauma and the connection to family and community members struggling with substance use issues. As a result, they abstain from alcohol, or they drink in moderation. The following participant’s reflections on the role of Native community and culture on alcohol use interwove both ideas in the context of the pandemic:

*So, I grew up very disconnected from my tribe. I’m the [tribe], and we’re the [descriptor] non-federally recognized tribe in the US. And I kind of spent my quarantine and isolation trying to reconnect because there was always this part of myself that I knew that I needed to have, and I needed to learn more of. And that’s kind of what I did over COVID. I decolonized and indigeneity and everything. But in terms of the drinking, I know the history. And I don’t know–it scares me, I guess, because I know that it’s kind of in my blood, this potential for danger and alcoholism and all that. But I don’t know. Yeah. A part of me was just like, “We’ll figure it out.”*

### 3.2. Sexual Health Choices

Some participants shared that the pandemic heavily affected their sexual choices. Some chose to be more sexually active due to the stressors of the pandemic or having more free time:

*It was very-the pandemic, it was just a time of struggle and everything like that. So it more led me to take riskier choices and not be precautious and really care about my health or my safety, things like that. Yeah. So that was during the pandemic.*

Others abstained from sex due to the fears of contracting COVID-19, as one participant reflected:

*[The pandemic] has greatly affected [choices about sex] because I know now there’s a pandemic, so I have to be extra careful about who I come in contact with. So, it’s really, really affected who I’m with, who I spend my time with.*

Other participants shared that the pandemic did not directly affect their sexual choices. Instead, growing older and the expectations and pressures of sex were reasons they chose to be less sexually active. In contrast, some participants shared that growing older caused them to understand themselves more and their relationship with sex and have a more positive and empowered view.

*[W]ith the pandemic, there’s been a lot more positivity towards sex and woman empowerment and talking of making sex a less taboo topic and talking about it. And I think that having that kind of positivity around sex is good-So I believe that if we all talk about it together, and we’re open and have those conversations it’s to kind of just open everyone up to the idea of [having sex].*

A couple of participants reported sexual identity transitions during the pandemic. One participant reflected that they felt objectified and fetishized in the past and now have a new perspective on relationships. They found someone who understood them and built a relationship that brought healing, as they describe how the pandemic affected their choices about sex:

*[The pandemic] affected choices about sex in many ways, I think. A few key ones would be this relationship is so different from any relationship that I’ve had in the past in that, before, I was with a lot of cisgender heterosexual White men who approach sex completely differently than my current partner. We both identify as nonbinary. We’re both queer … [having] a partner who is really conscious about sex, I’ve actually learned that it’s okay to receive pleasure and have autonomy in a sexual situation. And also my partner is also Indigenous. So being with somebody who is also Native has been really healing because I have really had to face how much of my past sexual relationships were contingent upon my fetishization and objectification.*

Participants described a range of experiences regarding contraception. Most participants shared that they were using birth control. Some had used birth control in the past and stopped, and some had never used birth control. Some participants reported stopping birth control due to negative side effects, such as weight gain and mood swings. Some shared that they had adequate access to birth control and resources during the pandemic—with one participant stating that the pandemic, in fact, made it easier to access birth control because Planned Parenthood had a program for 16-19-year-olds to acquire birth control for free. Some, however, noted they had a hard time accessing birth control due to limited provider appointment availability during the pandemic and the hospitals being overwhelmed.

### 3.3. Identity and Self-Reflection

Participants frequently shared how the pandemic and their time in isolation were a time of reflection and learning about their identities. Some participants shared how the pandemic gave them time to self-reflect and discover how they fit into the world. They reflected on how witnessing COVID-19 deaths among their families and communities affected their viewpoints on life and identities.

*I think I learned, yeah, definitely how to be present within my own self, how to formulate a relationship of self-trust and self-acceptance within who I am and embracing that. I’ve learned how to let go of shame and to let myself feel things like anger and rage and grief in a way that is healthy. And I’ve learned how to really come home to myself because I’m really young and I didn’t have really anybody-I haven’t had the opportunity to have a lot of healthy relationships, but learned that I can give that to myself. As long as I have a safe space, I can flourish and I can grow and I can learn how to feel safe-since the pandemic and since the last couple of years, I’ve been really just like setting the foundation for yeah, this deep embodiment and yeah, presence.*

Many reported learning to set realistic goals for their future while creating space to be in the present moment. One participant shared that they learned to forgive themselves for their past mistakes and, in the process, built better relationships with their family. One participant shared their process of becoming an adult and navigating that world:

*I guess throughout this whole time I’ve been learning a lot of things about how to, I guess, navigate the adult world, because I went through that transition during the pandemic. I had to learn how to pay rent, get an apartment, apply to college-all of that.*

Some participants talked extensively about how the pandemic caused them to look within and reevaluate their relationships related to parts of their lives like home life or career. In addition to finding their identities and self-reflection, some participants mentioned how they reconnected with their Native culture during this identity development stage. Participants mentioned reconnecting with their culture; one participant stated they would go out to the land to reconnect. Another participant shared that they moved to foster care during the pandemic and reconnected with their tribal culture. Reconnecting was a way to buffer the day-to-day difficulties during the pandemic by attending ceremonies and dances and engaging in more traditional practices.

### 3.4. Mental Health

Many of the participants shared how the pandemic negatively affected their mental health, but it also allowed for them to build coping tools and find healing. Many participants reflected that isolation due to stay-at-home restrictions during the pandemic’s beginning was taxing on their health. Isolation caused them to have less access to social situations and contact with others, as many adhered to stay-at-home orders to keep themselves and their families safe. They felt lonely, which caused anxiety and depression in some.

*So, at the beginning when we really couldn’t go outside, we really couldn’t socialize, I was pretty sad because I couldn’t see my friends or anyone at school or my family. Yeah. That was the biggest challenge. So, a lot of isolation.*

Despite these mental health struggles, many participants shared emotional and relational upsides to the pandemic. One participant shared how they used isolation to look inward and reflect on gratitude for their lives and those they love. Others shared how they learned to communicate better with their family, got in touch with their spirituality and culture, and found creative outlets such as playing musical instruments and making crafts with friends and family.

*[My set of coping skills] helps me process and sift through a lot of the internal stuff that I have going on and sort of get an idea of what’s coming up, what needs to be worked through, what are the obstacles that are preventing me from feeling safe and present, and how can I work through those things to arrive at a peaceful place? And body practices like during the pandemic, I’ve developed a lot of practices with yoga and breath work, meditation was big for me for a while, dancing, so just letting myself dance freely and move, creative stuff too like singing, dancing, making music, things like that.*

### 3.5. Relationship Dynamics

Differing vaccine beliefs and political turmoil during the COVID-19 pandemic strained some participants’ relationships with family and friends. Discussions around safety measures prompted confrontations and anxiety-provoking situations for navigating time with family and friends. Alienation and estrangement from loved ones exacerbated mental health issues for some. However, others shared how they built stronger relationships during the pandemic and spent time growing closer to loved ones:

*I feel like finding out things my friends and I could do or my boyfriend and I finding small things we could go do without COVID being a huge deal. That helped. [For example, my] boyfriend lives by one of the wetland preserve things. And so we were able to go out there and walk around and stuff like that because it was outside.*

Some also shared that they valued time spent with their family in quarantine and understood the importance of being close to their grandparents. One participant shared that they appreciated their parents checking in on them often and ensuring they spent quality time outside and stayed connected with their family and friends. Most participants had memorable stories about how they became closer to their loved ones and reflected on the necessity of building strong relationships during the challenging pandemic.

*I feel like I’ve found more value towards having the people I have around me, good people, and keeping people close to me because I feel like during the things that I’ve gone through, I would have thought I’d push away from people. But I’ve gotten really close to my brother. He’s one of my best friends. And I think it’s more trying to find that connecting point towards people and learning about how to forgive myself for the things that’s happened.*

## 4. Discussion

This qualitative study sought to understand urban Native young women’s experiences, behavior, and perceptions informing their ideas and actions regarding AEP risk during the COVID-19 pandemic. IDIs were conducted between March and May 2022, when lockdown requirements were easing despite the Omicron variant wave still peaking. These qualitative data shed light on participants’ changes from pre-pandemic life, coupled with assessing AEP risk-related behaviors within the context of the pandemic effects on health and community life. The original intent of the interviews was to determine behavior change in AEP risk, including alcohol use, sexual activity, and contraceptive use within the context of the COVID-19 pandemic. The purposive sample design allowed diverse perspectives from those with varying AEP risk profiles and COVID-19-related experiences.

Given changes in alcohol accessibility throughout the nation [43] coupled with pandemic-induced boredom and stress, it is not surprising that many participants reported increased drinking. However, some participants chose not to drink alcohol or to drink less. Sometimes, their reasons for abstaining from or reducing alcohol use were not closely related to the pandemic but were the result of ongoing family expectations, reactions to negative family history, or reflection of self and the relationship to cultural beliefs. Participants often described their alcohol use (or non-use) within the context of social relationships. Unlike other research, only one of the participants noted drinking alcohol with family members. Some participants also shared their choices to abstain from drinking due to their family’s history of alcoholism and the historical impacts of drinking on Native communities. Friends often influenced participants—in both positive and negative ways depending on whether they encouraged participants to drink or to abstain from alcohol. Others noted a personal preference that was not dictated by the influence of their friends, families, and communities but derived from self-reflection and a sense of self.

Sexual health choices for many people nationwide were affected by the COVID-19 pandemic with studies showing decreased activity due to social distancing and fear of contracting COVID-19 through close contact [44] and decreased sexual functioning even when frequency of activity did not change [45]. Participants in our study described similar reasoning for less sexual contact. However, some participants reported increased sexual activity due to the stressors of the pandemic and having more free time. Some had consistent partners and chose to spend time with them despite social distance orders. Participants were also conscious of their development into adulthood and how their sexual choices may not necessarily have been related to the COVID-19 pandemic. Some noted the ambiguity of their sexual experiences—whether they should be attributed to the pandemic or to maturing from adolescence into adulthood. This observation is astute—for these participants, the transition from adolescence to adulthood will be indelibly marked by the stressors, loss, and grief wrought by the pandemic. Notable is the fact that amid so many reporting mental health issues and stress from the uncertainty of day-to-day life, losing loved ones, and isolation, so many offered thoughtful and considered self-reflection, including drawing from and learning more about cultural values, practices, and beliefs. Participants reflected on the pressures and expectations of sex but also shared a more empowered vision of themselves and positive views of sex. Unlike our expectations of limited contraception availability during the pandemic, especially for youth, many participants reported adequate or even increased birth control access. However, some participants reported they could not get an appointment due to the pandemic and overwhelmed hospitals, which is true for others more broadly during the pandemic [46].

Participants commonly used the terms anxiety, isolation, and depression to describe their mental health during the pandemic; findings echoed in other research [47]. Despite these mental health struggles, our participants shared many coping skills they learned. They understood that even though they felt alone, everyone was struggling, and they were all in it together. Participants reflected that the pandemic reminded them to value time with friends and family and to be grateful for loved ones. They took the time to reach out to their friends and loved ones to build connections, go in nature, look inward for healing opportunities, and build more connections with their cultures and traditions. The pandemic gave them a chance to evolve and build their identities. They used it as an opportunity for self-reflection and growth.

These findings about urban Native young women’s AEP-related experience during the pandemic should be understood within study limitations. While the participants were selected purposively [38] to ensure the breadth and depth of AEP risk and COVID-19-related experiences were represented, the sample size was small relative to the entire population of Native youth. Though the sample size was within the range typical for achieving saturation based on a recent review of empirically based studies of qualitative sample sizes that found 9-17 interviews were sufficient [48], generalizing beyond this sample of 15 should be done cautiously—particularly considering the tremendous diversity of Native communities in this country, and as cautioned by Hansen et al. [49] specifically with respect to studies of AEP risk among Native populations. In addition, though all efforts were made to provide participants with privacy and confidentiality to encourage honesty, participants may nonetheless have altered their responses to increase social acceptability. Further, the interviews did not elicit information about the impact of structural racism or racial discrimination on young women’s experiences during the pandemic—factors known to have driven the disparities in COVID-19 morbidity and mortality experienced by Native populations and that may be unique in degree, kind, or influence within urban settings. Interviews also did not elicit young women’s perspectives on the importance of Native values surrounding pregnancy and childbirth, which can act as protective factors against AEP risk [50]. Despite these limitations, it is important to point out that like other qualitative studies, including those focused on understanding Native youth AEP risk [13], the sample was not designed to be representative of this population generally or to give population estimates, but to instead provide rich, in-depth context and nuance on experiences of this underserved and under-researched population. Such insights are important for informing healing processes, hypothesis development, and the ways in which future work can continue to fill the gaps in our understanding of how to address AEP risk and promote health, wellbeing, and thriving more broadly for Native youth.

## 5. Conclusions

The current study focused on a Native community often overlooked in research—urban communities—and AEP risk and resilience, a priority area for Native communities. Indeed, these data revealed that the pandemic influenced participants’ drinking, sexual activity, and contraceptive use behavior, though not in uniform or predictable ways, and not without reflection and strategies of self- and community connection. Participants shared their resilience, creativity, and strength in the face of adversity—these are stories of young wisdom rarely captured in the research literature but vital to supporting the health and wellness of this underserved community as it continues to heal.

## Figures and Tables

**Table 1:** Emergent themes regarding alcohol use, sexual health, identity, mental health, and relationships among urban Native young women during the pandemic.

**Patterns of Alcohol Use**
• Pandemic Isolation, poor mental health, boredom: increased use
• Alcohol easier to obtain: increased use
• Growing older, new contexts (e.g., college)
• Family/friends influences both positive and negative
• Role of self-efficacy in choosing to drink or not to drink
• Role of Native values regarding alcohol use: decreased use
• Historical trauma impacts shaping alcohol use patterns in family, community
**Sexual health choices**
• Stressors of pandemic, more free time: increased sex
• COVID-19 transmission fears: decreased sex
• Growing older, changing understanding of self–empowered and positive
• Time of sexual identity transitions
• Access to contraception during the pandemic varied
**Identity and self-reflection**
• Isolation brought on self-reflection
• Witnessing COVID-19 deaths affected viewpoints on life and identities
• Worked on improved relationships with family
• Reconnection with culture—ceremonies, dances, and more traditional practices to buffer pandemic impacts
**Mental health**
• Pandemic brought on isolation, loneliness, anxiety, depression
• Built coping tools and find healing: self-forgiveness, gratitude, spirituality, creativity
**Relationship dynamics**
• Strained personal relationship regarding vaccine beliefs, political turmoil, masking, lockdowns
• Valued time with family in quarantine
• Gratitude for parents, grandparents, community
• Importance of strong relationships

## Data Availability

While all interviews were de-identified, they contain highly sensitive information, and it is possible content could be recognized and attributed to a participant. Thus, we do not provide public access to these data. We invite interested researchers to contact the Principal Investigators for further information.
